# Impact of Gestational Diabetes on Neonatal Birth Weight and Maternal Postpartum Metabolic Changes

**DOI:** 10.7759/cureus.86060

**Published:** 2025-06-15

**Authors:** Aqsa Arbab Khan, Saba Javed, Sobia Noreen, Muhammad Raheel Chaudhry, Sanodia Afridi

**Affiliations:** 1 Obstetrics and Gynaecology, Letterkenny University Hospital, Letterkenny, IRL; 2 Obstetrics and Gynaecology, Health Net Hospital, Peshawar, PAK; 3 Internal Medicine, Letterkenny University Hospital, Letterkenny, IRL; 4 Medicine, Health Net Hospital, Peshawar, PAK

**Keywords:** gestational diabetes, insulin resistance, macrosomia, maternal health, neonatal outcomes, postpartum metabolism

## Abstract

Background: Gestational diabetes mellitus (GDM) is a growing public health concern globally, particularly in the context of rising maternal obesity and glucose intolerance. GDM poses significant risks to both neonatal and maternal health, including fetal overgrowth, birth complications, and long-term metabolic disorders. This study aimed to evaluate the impact of GDM on neonatal birth weight and maternal postpartum metabolic changes. The primary objective was to assess the incidence of macrosomia and small-for-gestational-age (SGA) births in pregnancies complicated by GDM. Secondary objectives included evaluating postpartum glycemic and lipid profiles, comparing metabolic parameters at six weeks and six months between GDM and non-GDM groups, and identifying predictive and moderating variables such as glycemic control, pre-pregnancy BMI, and gestational weight gain.

Methodology: A prospective cohort study was conducted over an 18-month period (October 2023 to April 2025) at Health Net Hospital, a tertiary care and referral center located in Peshawar, Pakistan. A total of 219 pregnant women were initially screened for eligibility. Following the application of inclusion and exclusion criteria, 189 women were enrolled, 94 diagnosed with GDM and 95 without GDM, serving as matched controls. Data were collected at three time points: during the second trimester (baseline), at delivery, and during postpartum follow-up at six weeks and six months. Maternal metabolic markers, including glycemic and lipid profiles, were assessed longitudinally, while neonatal outcomes such as birth weight, NICU admission, and hypoglycemia were documented at birth. Follow-up adherence was ensured through scheduled reminders and flexible appointment rescheduling. Clinical and laboratory data were collected using standardized protocols by trained staff. Logistic regression analysis was used to identify independent predictors of adverse maternal and neonatal outcomes, with adjustments for potential confounders. Data analysis was performed using IBM SPSS Statistics for Windows, Version 29.0.2.0 (IBM Corp., Armonk, New York, United States).

Results: Neonates of GDM mothers had significantly higher birth weights (mean 3689 g vs. 3143 g; p<0.0001), with increased incidence of neonatal hypoglycemia in 16 (17%) GDM cases compared to four (4.2%) non-GDM cases and NICU admissions in 19 (20.2%) GDM neonates versus five (5.3%) non-GDM neonates. GDM mothers exhibited elevated fasting glucose at six weeks (mean 98.5 mg/dL) and six months (94.1 mg/dL) postpartum, compared to non-GDM mothers (85.3 mg/dL and 83.2 mg/dL, respectively; p<0.0001). Homeostasis Model Assessment of Insulin Resistance (HOMA-IR) and triglyceride levels were also significantly higher in the GDM group. GDM (OR=2.8), higher pre-pregnancy BMI, and poor glycemic control (HbA1c >5.9%) were independent predictors of neonatal macrosomia and maternal insulin resistance.

Conclusion: GDM significantly increases the risk of macrosomia, neonatal complications, and sustained maternal metabolic dysfunction postpartum. These findings underscore the importance of early diagnosis, effective glycemic control, and structured postpartum follow-up to mitigate long-term risks.

## Introduction

Gestational diabetes mellitus (GDM) is a glucose intolerance that arises during pregnancy, affecting approximately 7-14% of pregnancies globally, with even higher rates in South Asia [1,2]. GDM poses considerable risks to both maternal and neonatal health, including preeclampsia, macrosomia, cesarean delivery, and the future development of type 2 diabetes in mothers [3]. In neonates, the consequences may include birth trauma, hypoglycemia, and long-term metabolic derangements [4]. As the global burden of GDM rises, especially in low- and middle-income countries, it becomes increasingly essential to understand its impact on short- and long-term outcomes. While distinct from type 1 diabetes (T1D), which is a complex autoimmune illness influenced by epigenetic, environmental, and hereditary factors, both conditions underscore the systemic nature of glycemic dysregulation in different life stages [5]. An awareness of these underlying connections is essential for developing effective prevention and management strategies across the spectrum of diabetes. GDM, in particular, poses considerable risks to both maternal and neonatal health.

Numerous studies have documented the association between GDM and adverse neonatal outcomes, particularly macrosomia (birth weight ≥4000 g) and small-for-gestational-age (SGA) births (birth weight <10th percentile for gestational age), both of which are predictors of perinatal complications and future metabolic syndrome [6-8]. Moreover, there is growing recognition that the effects of GDM do not end at delivery. Postpartum glucose intolerance and persistent insulin resistance have been reported in women with prior GDM, yet the metabolic trajectories during the early postpartum period remain inadequately characterized [9,10].

Although clinical guidelines emphasize postpartum follow-up, the implementation remains suboptimal, and data on the dynamic recovery or progression of maternal metabolism over the initial six months postpartum are scarce. Most existing studies fail to integrate moderating variables such as pre-pregnancy BMI, gestational weight gain, and mode of delivery, all of which may influence both neonatal birth weight and postpartum metabolic profiles. This gap in literature is especially evident in high-burden but under-researched regions like Peshawar, Pakistan, where cultural, nutritional, and healthcare access differences may impact outcomes.

This study aimed to evaluate the impact of GDM on neonatal birth weight and maternal postpartum metabolic changes. The primary objective was to assess the incidence of macrosomia and SGA births in pregnancies complicated by GDM. The secondary objectives included evaluating postpartum glycemic and lipid profiles, comparing metabolic parameters at six weeks and six months between GDM and non-GDM groups, and identifying predictive and moderating variables such as glycemic control, pre-pregnancy BMI, and gestational weight gain.

## Materials and methods

Ethical considerations

Prior to initiating the study, ethical clearance was obtained from the Ethics Review Committee of Health Net Hospital (HNH), Peshawar, under reference number 3061/HNH/HR. All participants received written and verbal information regarding the study objectives, procedures, and voluntary nature of participation. Written informed consent was secured from each subject in compliance with the Declaration of Helsinki. Participant confidentiality was ensured through anonymized data handling.

Study design and setting

A prospective observational cohort study was conducted at the Department of Obstetrics and Gynaecology, HNH, Peshawar. HNH is a major tertiary care teaching hospital and referral center in Khyber Pakhtunkhwa province, serving a diverse and high-volume patient population. The study was carried out over a total duration of 18 months from October 2023 to April 2025, allowing sufficient time for recruitment, delivery, and postpartum follow-up of all participants.

Study population

Pregnant women aged between 18 and 45 years with singleton pregnancies who were attending routine antenatal care at HNH were screened during their second trimester. Those diagnosed with GDM using the International Association of Diabetes and Pregnancy Study Groups (IADPSG) criteria [11] were included in the exposed group. The control group consisted of age-, BMI-, and parity-matched pregnant women who did not develop GDM. Women with known pre-existing diabetes mellitus, multiple pregnancies, chronic hypertension, thyroid dysfunction, or renal disorders or those who failed to complete postpartum follow-up were excluded.

Sample size and sampling method

The participant flow of the study began with 219 pregnant women screened for eligibility at HNH, Peshawar. Of these, 189 met the inclusion criteria and were deemed eligible, while 30 were excluded: 20 due to not meeting the criteria and 10 who declined participation. All 189 eligible participants were successfully enrolled and allocated into two groups: 94 diagnosed with GDM and 95 as non-GDM controls. All participants proceeded through delivery, and postpartum follow-up was conducted. At six months postpartum, 94 participants from the GDM group and 95 from the non-GDM group completed the study, resulting in a final analyzed sample of 189 women. This complete retention reflects excellent follow-up adherence and data integrity across both cohorts.

Data collection procedures

Data collection occurred at three primary time points: (1) during the second trimester (baseline), (2) at the time of delivery, and (3) during postpartum follow-up visits at six weeks and six months. Maternal sociodemographic information, pre-pregnancy weight, gestational weight gain, parity, and glycemic indicators such as fasting glucose, HbA1c, and oral glucose tolerance test (OGTT) results were recorded at baseline.

Maternal biochemical markers, including serum insulin, fasting glucose, triglycerides, cholesterol, and Homeostasis Model Assessment of Insulin Resistance (HOMA-IR), were measured at both postpartum intervals. Blood samples were analyzed at the hospital's central laboratory using standardized enzymatic assays (Abbott Architect c8000 system, Chicago, Illinois, United States) following the manufacturer's protocols. All laboratory instruments underwent regular calibration using internal quality controls and external standards traceable to the National Institute of Standards and Technology (NIST).

Neonatal outcomes, including birth weight, gestational age, Apgar scores, and complications such as macrosomia, hypoglycemia, or NICU admission, were documented at delivery. Macrosomia was defined as birth weight ≥4000 g, and SGA was defined as birth weight below the 10th percentile for gestational age based on INTERGROWTH-21st standards [12]. All data were collected using standardized tools and calibrated equipment by trained clinical staff blinded to group assignments during neonatal assessments.

Follow-up monitoring and patient feedback

Participants were reminded of follow-up appointments at six weeks and six months postpartum through telephone calls and SMS. Compliance was recorded, and efforts were made to accommodate rescheduling. A short anonymous feedback form was administered at the six-month visit to assess satisfaction with care and participation experience. A summary of the follow-up is given in Table 1.

**Table 1:** Follow-up duration

Phase	Estimated time
Recruitment period	6-9 months
Pregnancy monitoring	~6 months (during ongoing recruitment)
Postpartum follow-up	6 months after the last delivery
Data cleaning and analysis	1-2 months

Variables and outcome measures

The primary neonatal outcome was birth weight, classified as normal, macrosomia (≥4000 g), or SGA (<10th percentile). The primary maternal outcomes included insulin resistance, glucose intolerance, and altered lipid profiles observed during the postpartum follow-up periods. Secondary neonatal outcomes included Apgar score <7 at five minutes, hypoglycemia, and NICU admission. Secondary maternal outcomes included postpartum weight retention and risk of postnatal depression measured using the Edinburgh Postnatal Depression Scale.

Data quality and bias control

Selection bias was minimized through the use of consecutive sampling. Confounding variables such as maternal age, BMI, gestational weight gain, and delivery mode were accounted for through matching and multivariable regression modeling. Data collectors were blinded to group status during neonatal evaluation to reduce information bias. Standardized operating procedures and laboratory protocols were employed to ensure measurement consistency. Missing data were addressed using multiple imputation techniques, and follow-up loss was documented systematically.

Subgroup and sensitivity analysis

Subgroup analyses were conducted based on GDM management strategy (diet-controlled vs. insulin-treated), pre-pregnancy BMI classification, and timing of diagnosis. Sensitivity analyses excluded participants who developed gestational hypertension or had incomplete biochemical follow-up to validate the robustness of findings.

Statistical analysis

Statistical analyses were conducted using IBM SPSS Statistics for Windows, Version 29.0.2.0 (IBM Corp., Armonk, New York, United States), beginning with the calculation of descriptive statistics for all baseline variables. Subsequently, continuous variables were assessed using independent t-tests and Mann-Whitney U tests based on their distribution, while categorical variables were analyzed via chi-squared tests. Multivariate logistic regression was utilized to control for potential confounding factors. Statistical significance for all tests was defined as a p-value less than 0.05.

## Results

Women with GDM exhibited significantly different metabolic profiles during pregnancy compared to non-GDM individuals (Table 2), characterized by a significantly higher pre-pregnancy BMI (30.19±3.25 vs. 26.32±2.65; p<0.0001), elevated fasting glucose levels (100.01±9.98 mg/dL vs. 85.58±7.23 mg/dL; p<0.0001), and higher HbA1c values (5.87 vs. 5.31; p<0.0001), indicative of poorer glycemic control, despite similar maternal age and parity (p>0.3); furthermore, while triglyceride levels showed a trend towards significance (p=0.068), high-density lipoprotein (HDL) levels were significantly lower in the GDM group (44.55±5.85 mg/dL vs. 46.55±5.76 mg/dL; p=0.0159), suggesting early dyslipidemia and highlighting the distinct metabolic baseline of women with GDM during pregnancy.

**Table 2:** Baseline characteristics and pregnancy glycemic profile GDM: gestational diabetes mellitus; HDL: high-density lipoprotein; LDL: low-density lipoprotein U: Mann-Whitney test values; t: t-tests; *: statistically significant (p<0.05)

Variable	GDM (mean±SD) (n=94)	Non-GDM (mean±SD) (n=95)	Test statistic	P-value
Age (years)	29.58±3.64	30.09±3.82	U=420	0.3423
Pre-pregnancy BMI (kg/m²)	30.19±3.25	26.32±2.65	t=9.21	<0.0001*
Parity	1.29±1.04	1.46±1.12	U=445	0.2675
Gestational weight gain (kg)	11.68±2.98	12.08±3.21	t=-0.9	0.3716
Fasting glucose (mg/dL)	100.01±9.98	85.58±7.23	t=11.71	<0.0001*
HbA1c (%)	5.87±0.28	5.31±0.20	t=16.39	<0.0001*
Triglycerides (mg/dL)	165.29±26.96	158.64±24.19	U=420	0.068
HDL (mg/dL)	44.55±5.85	46.55±5.76	t=-2.43	0.0159*
LDL (mg/dL)	110.51±15.99	110.99±15.30	t=-0.22	0.8285

Significant differences were observed in neonatal outcomes between the GDM and non-GDM groups (Table 3). Neonates born to mothers with GDM had significantly higher birth weights (3689.1±423.4 g vs. 3142.8±367.3 g; p<0.0001), although the incidence of macrosomia (25% vs. 15%) did not reach statistical significance (p=0.1109). Other adverse outcomes, such as neonatal hypoglycemia (17% vs. 4%; p=0.0046) and NICU admissions (20% vs. 5%; p=0.0022), were significantly more frequent in the GDM group. Apgar scores <7 at five minutes were higher among GDM infants (7% vs. 2%), but not statistically significant. Independent t-tests were used for continuous variables, yielding a t-value of 9.31 for birth weight and 0.50 for gestational age at delivery, while chi-squared tests were applied for categorical outcomes, including macrosomia (χ²=2.53), SGA (χ²=0.00), Apgar score <7 (χ²=2.38), neonatal hypoglycemia (χ²=8.06), and NICU admission (χ²=9.66). These findings reflect the increased neonatal risk profile associated with maternal hyperglycemia.

**Table 3:** Neonatal outcomes by maternal GDM status GDM: gestational diabetes mellitus; *: statistically significant (p<0.05) t-tests were used for continuous variables (e.g., birth weight, gestational age). Chi-squared tests (χ²) were used for categorical outcomes.

Outcome	GDM (n=94)	Non-GDM (n=95)	Test statistic	P-value
Birth weight (g)	3689.1±423.4	3142.8±367.3	t=9.31	<0.0001*
Gestational age at delivery (weeks)	38.56±1.21	38.47±1.19	t=0.50	0.6141
Macrosomia (≥4000 g)	24 (25.5%)	14 (14.7%)	χ²=2.53	0.1109
Small for gestational age	8 (8.5%)	8 (8.4%)	χ²=0.00	1.0000
Apgar score <7 at 5 minutes	7 (7.4%)	2 (2.1%)	χ²=2.38	0.1697
Neonatal hypoglycemia	16 (17%)	4 (4.2%)	χ²=8.06	0.0046*
NICU admission	19 (20.2%)	5 (5.3%)	χ²=9.66	0.0022*

At both six weeks and six months postpartum, women in the GDM group exhibited persistently elevated metabolic markers compared to their non-GDM counterparts (Table 4). The mean fasting glucose in the GDM group declined from 98.5 mg/dL at six weeks to 94.1 mg/dL at six months, yet remained significantly higher than the non-GDM group (85.3 and 83.2 mg/dL, respectively; p<0.0001). Similarly, insulin resistance (HOMA-IR) remained higher in GDM mothers (3.1 and 2.8) than in non-GDM counterparts (2 and 1.8) across both time points (p<0.0001). Triglyceride levels, low-density lipoprotein (LDL) values, and HDL values followed a similar trend, demonstrating sustained metabolic dysregulation in the postpartum period for women with prior GDM.

**Table 4:** Maternal postpartum metabolic changes (six weeks and six months) GDM: gestational diabetes mellitus; HOMA-IR: Homeostasis Model Assessment of Insulin Resistance; HDL: high-density lipoprotein; LDL: low-density lipoprotein *: statistically significant (p<0.05); t: t-tests

Parameter	GDM (mean±SD) (n=94)	Non-GDM (mean±SD) (n=95)	t-value	P-value*
Fasting glucose (mg/dL): 6 weeks	98.5±8.2	85.3±6.5	12	<0.0001
Fasting glucose (mg/dL): 6 months	94.1±7.8	83.2±6.1	8.52	<0.0001
HOMA-IR: 6 weeks	3.1±0.9	2.0±0.7	8.26	<0.0001
HOMA-IR: 6 months	2.8±0.8	1.8±0.6	9.54	<0.0001
Triglycerides (mg/dL): 6 weeks	175.2±26.1	160.8±21.5	5.67	<0.0001
Triglycerides (mg/dL): 6 months	169.3±24.7	158.1±20.9	4.64	<0.0001
HDL (mg/dL): 6 weeks	44.1±5.4	47.5±5.2	-3.08	0.0023
HDL (mg/dL): 6 months	45.3±5.3	48.8±5.4	-5.18	<0.0001
LDL (mg/dL): 6 weeks	115.4±14.9	109.2±13.7	2.97	0.0033
LDL (mg/dL): 6 months	112.7±15.1	107.6±13.3	3.88	0.0001

Multivariate logistic regression in Table 5 revealed that GDM significantly increased the odds of neonatal macrosomia (OR=2.8; 95% CI: 1.5-5.3) and postpartum insulin resistance (OR=3.4; 95% CI: 1.9-6.0). Other independent predictors included pre-pregnancy BMI (OR=1.12 for macrosomia; OR=1.15 for HOMA-IR >2.5) and gestational weight gain. Poor glycemic control (HbA1c >5.9%) was strongly associated with both outcomes (macrosomia OR=1.85; HOMA-IR OR=2.1). Cesarean delivery was not found to be a significant predictor. These findings reinforce the multifactorial nature of adverse neonatal and maternal outcomes associated with GDM.

**Table 5:** Multivariate logistic regression: predictors of neonatal macrosomia and maternal insulin resistance GDM: gestational diabetes mellitus; HOMA-IR: Homeostasis Model Assessment of Insulin Resistance The statistical analyses included independent t-tests for continuous variables, chi-squared tests (χ²) for categorical variables, and multivariate logistic regression to assess predictors of neonatal macrosomia and maternal insulin resistance.

Predictor	Adjusted OR (macrosomia)	95% CI	Adjusted OR (HOMA-IR >2.5)	95% CI
GDM (yes vs. no)	2.8	1.5-5.3	3.4	1.9-6.0
Pre-pregnancy BMI (per kg/m²)	1.12	1.03-1.21	1.15	1.05-1.26
Gestational weight gain (per kg)	1.09	1.01-1.17	1.05	0.98-1.12
Poor glycemic control (HbA1c >5.9%)	1.85	1.1-3.2	2.1	1.3-3.5
Cesarean delivery	1.02	0.6-1.7	1.22	0.8-1.9

Among the 94 women with GDM, 60 (63.8%) were managed with dietary modifications alone (diet-controlled GDM), while 40 (36.2%) required insulin therapy (insulin-treated GDM). When stratified by treatment modality, insulin-treated GDM mothers had higher neonatal birth weights (3760±420 g vs. 3520±400 g; p=0.0119) and higher HOMA-IR values at six months (3.4±0.7 vs. 2.5±0.6; p<0.0001) compared to diet-controlled GDM mothers (Table 6). While rates of macrosomia and NICU admission were higher in the insulin group (32.5% and 27.5%, respectively), these differences were not statistically significant. This suggests that insulin-requiring GDM may reflect more severe disease and warrants closer follow-up.

**Table 6:** Comparison of neonatal and maternal outcomes between diet-controlled and insulin-treated GDM groups GDM: gestational diabetes mellitus; HOMA-IR: Homeostasis Model Assessment of Insulin Resistance *: statistically significant (p<0.05); t: t-tests; χ²: chi-squared tests

Parameter	Diet-controlled GDM (n=60)	Insulin-treated GDM (n=40)	Test statistic	P-value
Neonatal birth weight (g)	3520.0±400.0	3760.0±420.0	t=–2.57	0.0119*
Postpartum HOMA-IR (6 months)	2.5±0.6	3.4±0.7	t=–6.18	<0.0001*
Macrosomia (≥4000 g)	12 (20%)	13 (32.5%)	χ²=1.91	0.1671
NICU admission	9 (15%)	11 (27.5%)	χ²=2.23	0.1359

## Discussion

The comparison of baseline characteristics revealed distinct metabolic profiles between women diagnosed with GDM and those without. GDM participants generally presented with higher pre-pregnancy BMI and elevated glycemic markers such as fasting glucose and HbA1c, even in the absence of significant age or parity differences. While overall gestational weight gain was similar between the two groups, GDM mothers demonstrated lower HDL and modestly higher triglyceride levels, suggesting early manifestations of insulin resistance and lipid dysregulation.

These findings align with existing literature that associates GDM with pre-existing metabolic dysfunction. Catalano et al. described increased adiposity and insulin resistance in early pregnancy among women who later developed GDM [13]. The beta-cell dysfunction combined with obesity-associated insulin resistance contributes to the pathophysiology of GDM [14]. The observed lipid alterations are also consistent with a study that reported altered lipid profiles in GDM pregnancies, emphasizing their role as potential biomarkers of metabolic derangement [15].

GDM was associated with a noticeable shift in neonatal outcomes, with infants more likely to exhibit higher birth weights, increased risk for macrosomia, and greater neonatal morbidity. Although not all differences reached statistical significance, trends suggested that maternal glycemic status has a substantial influence on fetal growth. In particular, neonatal hypoglycemia and NICU admissions were more common among infants born to mothers with GDM, reflecting the challenges of neonatal adaptation to an altered intrauterine environment.

These observations are supported by the Hyperglycemia and Adverse Pregnancy Outcome (HAPO) study, which demonstrated a continuous relationship between maternal glucose levels and adverse neonatal outcomes, even in glucose ranges below the diagnostic threshold for diabetes [16]. Similarly, studies by Ladfors et al. [17] and Hufnagel et al. [18] emphasized that fetal overgrowth is primarily driven by maternal hyperglycemia and insulin resistance, regardless of gestational age or parity. This underscores the importance of glycemic control not only for maternal outcomes but also for neonatal safety.

Postpartum follow-up at six weeks and six months revealed persistent metabolic alterations among women with prior GDM. These included elevated fasting glucose levels, higher insulin resistance (as assessed by HOMA-IR), and continued lipid abnormalities compared to the non-GDM cohort. While some improvements were observed over time, GDM mothers failed to return to metabolic baselines comparable to their non-GDM counterparts.

These findings mirror those reported by Miao et al. [19], who observed prolonged insulin resistance and impaired beta-cell function postpartum in women with prior GDM. Sheiner [20] and Lu et al. [21] in their studies found that GDM significantly increases the long-term risk of type 2 diabetes, reinforcing the need for continued surveillance. This study highlights the inadequacy of a "return to normal" assumption following delivery and emphasizes the postpartum period as a critical opportunity for metabolic risk modification.

Multivariate regression analysis identified GDM status, pre-pregnancy BMI, and suboptimal glycemic control (as indicated by elevated HbA1c) as significant predictors of both neonatal macrosomia and postpartum maternal insulin resistance. Gestational weight gain also showed a positive correlation with risk, though it appeared less influential than baseline BMI or glycemic markers.

These findings resonate with reports by Prakash et al. [22], who found that intensive glucose control during pregnancy reduced adverse neonatal outcomes. Both maternal adiposity and glycemia were found to contribute significantly to perinatal outcomes and long-term maternal health risks, as also stated by other studies [18,23], highlighting their dual impact on the trajectory of metabolic complications. The recognition of these variables as modifiable risk factors presents valuable opportunities for targeted intervention both during and after pregnancy.

In subgroup analysis, women with insulin-requiring GDM displayed more severe metabolic profiles postpartum and were more likely to have neonates requiring specialized care. Although these differences did not always achieve statistical significance, the trends indicate that insulin-requiring cases may represent a more severe GDM phenotype with less responsive beta-cell reserve.

This is supported by Anthony et al. [24], who reported higher complication rates in insulin-treated GDM, and Gupta et al. [25], who suggested that insulin therapy may be a marker of disease severity rather than simply a treatment modality. These findings underscore the importance of stratifying GDM not only by diagnosis but also by treatment type and degree of metabolic disturbance.

Strengths, limitations, and future perspectives

A key strength of this study lies in its prospective design, dual time-point follow-up, and inclusion of both maternal and neonatal outcomes, providing a more holistic understanding of GDM's short- to medium-term effects. Additionally, the use of biochemical markers and multivariable models enhances the robustness of its findings.

However, the study has limitations. Being single-centered limits generalizability to broader populations. Loss to follow-up and reliance on hospital-based care may introduce selection bias. Furthermore, lifestyle and dietary data, which are relevant to postpartum metabolic health, were not included. This allowed for a more focused analysis of biochemical and clinical outcomes directly attributable to GDM.

Future studies should involve multicenter collaboration to improve the representativeness and generalizability of findings across diverse settings and incorporate longer-term follow-up (e.g., 1-5 years postpartum), assess psychosocial factors such as postpartum depression and breastfeeding, and explore culturally appropriate intervention models. There is also a growing need to integrate digital tools such as mobile health platforms for remote follow-up and diabetes risk screening among women with prior GDM, particularly in low-resource settings.

## Conclusions

GDM significantly increases the risk of adverse neonatal outcomes, including macrosomia, neonatal hypoglycemia, and NICU admission, as well as sustained maternal metabolic dysfunction postpartum. These findings highlight that women with GDM remain metabolically vulnerable even up to six months after delivery. The results underscore the critical importance of early diagnosis, effective glycemic control during pregnancy, and structured, long-term postpartum follow-up. Implementing targeted lifestyle interventions and monitoring can play a vital role in improving both neonatal health and maternal metabolic recovery.
